# From Circularity to Spirality: An Integrated, Systems-Level Approach to Address the Plastics Problem

**DOI:** 10.1021/jacs.5c05454

**Published:** 2025-08-26

**Authors:** Patritsia M. Stathatou, Christos E. Athanasiou, Matthew J. Realff

**Affiliations:** † School of Chemical & Biomolecular Engineering, 1372 Georgia Institute of Technology, Atlanta, Georgia 30332, United States; ‡ Daniel Guggenheim School of Aerospace Engineering, 423866 Georgia Institute of Technology, Atlanta, Georgia 30332, United States; § Renewable Bioproducts Institute, Georgia Institute of Technology, Atlanta, Georgia 30332, United States

## Abstract

Plastics offer significant benefits but pose growing environmental and health concerns due to low recycling rates and continued reliance on fossil-derived feedstocks. While plastics circularity has emerged as a key strategy to reduce plastic waste and impacts, current mechanical recycling pathways face major limitations in maintaining material quality over repeated cycles. Advanced methods like chemical recycling and dissolution show promise but raise questions about environmental impacts, scalability, and cost. In this perspective, we introduce the concept of spirality as a more realistic model than perfect circularity, acknowledging the inevitable degradation of plastic quality over consecutive recycling cycles and the need for tiered recycling strategies. We emphasize the importance of early stage integration of mechanical property benchmarking, life cycle assessment (LCA), and techno-economic analysis (TEA) to evaluate emerging chemistry-enabled solutions for plastics recycling. In parallel, we underscore the critical role of high-quality data, and the need for multidisciplinary collaboration to align chemistry, materials science, engineering, systems analyses, and policy for sustainable transitions. Spirality, combined with robust assessment frameworks, can guide innovation toward more pragmatic and sustainable solutions in polymer design and end-of-life management.

## Graphical abstract

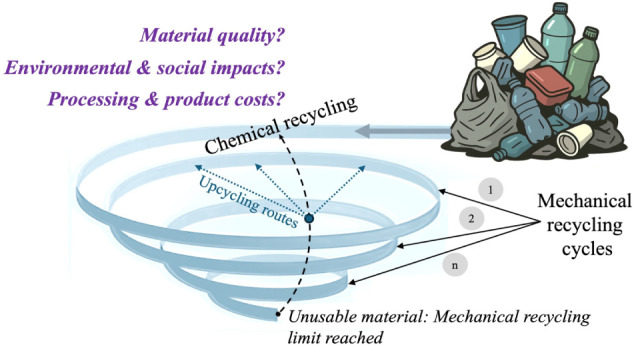

## 1 Introduction: Plastics—The Blessing, the Curse, and Circularity as a Potential Solution

Plastic products offer a unique combination of advantages, including versatility, durability, cost-effectiveness, and lightweight properties.^1^ As a result, they have dominated various industries, such as packaging, construction, and transportation. Innovations in plastics have also led to life-saving advancements in healthcare. Since the 1950s, their widespread benefits have driven the rapid plastic expansion, with global production of fossil-derived plastics reaching over 413 million metric tons (Mt) in 2023.^2^ This figure is projected to more than double by 2050.^3^

The vast majority of plastics production—about 360 Mt^4^—ends up as waste each year. According to United Nations’ data^5^ for 2023, 46% of plastic waste worldwide is landfilled, 22% is not properly disposed of ending up in the environment, 17% is incinerated and 15% is collected for recycling, with less than 9% being actually recycled after losses. In the United States (US), approximately 59 Mt of polymer resins were produced in 2023.^6,7^ Of this total, only 13% consisted of thermosets, while the remaining vast majority were thermoplastics.^7^ The mass flows of the most widely produced thermoplastics—accounting for about 80% of total polymer resin production in the US—from production through end-of-life are illustrated in Figure 1. Among the total thermoplastic waste generated in the US, about 73% ends up in landfills, 15% is incinerated with energy recovery, 8% is sent to recycling facilities,^8^ while about 2–3% is mismanaged,^9^ through litter or illegal dumping, and 1–2% is exported.^10^ Plastic containers and packaging make up the largest share of recycled plastics, accounting for 64% of the total US recycling stream.

**Figure 1:**
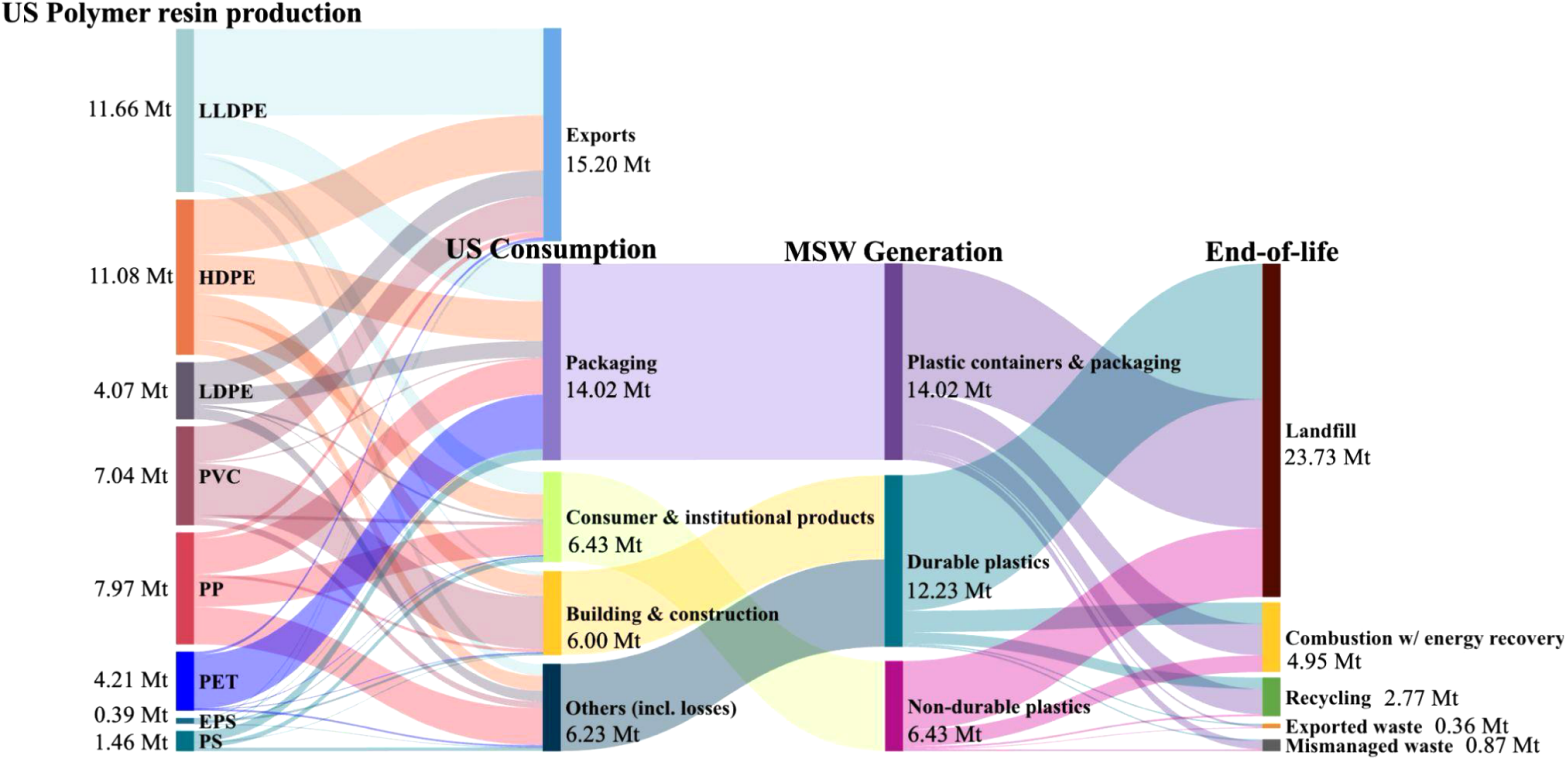
Mass flows of the most widely produced thermoplastics in the US in million metric tons (Mt) from production through end-of-life. Resin production and consumption data from ACC, 2024 ^7^ were used for 2023, except for PET, where production and consumption data from Smith et al., 2022,^11^ U.S. International Trade Commission, 2022,^12^ and Mordor Intelligence, 2024 ^13^ were used for 2022. Consumption includes domestic sales, captive use, and imports. Municipal solid waste (MSW) and end-of-life values follow the trends and categorization of US EPA, 2020.^8^ Mismanaged waste flows were estimated assuming the same litter and illegal dumping percentages reported by Law et al., 2020,^9^ while exported plastic waste flows were based on data from the Basel Action Network for 2023.^10^ Other thermoplastics not shown in the figure account for 3.35 Mt, and thermosets contribute an additional 7.80 Mt, bringing total US polymer resin production in 2023 to about 59.03 Mt^7^. LLDPE: Linear Low-Density Polyethylene; HDPE: High-Density Polyethylene; LDPE: Low-Density Polyethylene; PVC: Polyvinyl Chloride; PP: Polypropylene; PET: Polyethylene Terephthalate; EPS: Expanded Polystyrene; PS: Polystyrene.

Annual greenhouse gas (GHG) emissions from the plastic lifecycle, from extraction to manufacturing to end-of-life management, have reached nearly 2 billion tons of CO_2_-equivalent (CO_2_-eq),^14,15^ accounting for approximately 4% of the global GHG emissions.^3^ By 2040, plastics could account for up to 19% of global GHG emissions under a business-as-usual scenario.^3^ Currently, end-of-life emissions make up about 10% of the total plastic lifecycle emissions, primarily due to incineration, as plastics in landfills produce minimal GHG emissions (plastics take over 400 years to decompose), and recycling, as detailed above, handles only a small fraction of plastic waste.^14,16^ While landfilling plastics results in relatively low GHG emissions compared to organic waste, it poses other significant environmental and health risks. These include leaching of microplastics,^17^ release of chemical additives into soil and groundwater,^18^ and the potential generation of toxic emissions in the event of landfill fires.^19^ Unmanaged plastic waste that accumulates in the environment also generates emissions and poses environmental risks as it degrades in marine and terrestrial ecosystems,^20,21^ though efforts to quantify these emissions and impacts are still in early stages.

With approximately 90% of plastic lifecycle GHG emissions stemming from production, i.e., extraction, processing, and manufacturing stages,^14,16^ and over 98% of plastics currently derived from fossil sources,^16^ plastics circularity is considered a key solution to address waste accumulation, pollution, and emissions from virgin plastic production. Circularity, which involves recovering plastics after their end-of-life to create new products,^22^ is an important environmental goal. However, recycling the same carbon over and over for the same purpose is not always feasible, while in some cases, doing so may require additional resources, potentially outweighing the environmental benefits.

## 2 Plastics Circularity: The False Dichotomy of Closed- and Open-Loop Recycling

Acknowledging the well-established hierarchy of reduce, reuse and recycle,^5,8,23^ designing products for longevity and reusing plastics as much as possible should be the highest priority.^23^ However, these strategies are often impractical for single-use and packaging products, despite the growing interest in reusable packaging solutions.^16^ Given that the majority of plastic waste originates from single-use products,^1^ this perspective focuses on recycling approaches that promote plastics for material rather than product reuse, and particularly thermoplastic polymers.

Recycling of thermoplastics is often framed in terms of closed-loop versus open-loop systems, where the former refers to recycling plastics back into the same or similar products, and the latter involves converting them into different products, often of lower performance or quality.^23^ We argue that this binary classification oversimplifies the complexity of plastic flows and does little to advance sustainability goals. It presents a false dichotomy. In reality, the boundary between these categories is fluid, and the emphasis should be on maintaining material functionality, regardless of whether the recycled product is identical to the original.

To that end, we propose a more nuanced, integrated and flexible approach to plastics recycling and use, that we call spirality. Spirality transcends the closed/open dichotomy and embraces the role of chemistry and processing innovations in maintaining plastics within a continuous system of use for as long as possible with the highest quality. Circularity can be seen as the two-dimensional projection of a spiral, where loops created by use and recycling that degrade material quality are connected by operations that improve or maintain material quality.

Building on the concept of spirality, we next analyze the main recycling pathways currently in use, i.e., mechanical, chemical, and dissolution-based recycling,^24–27^ and examine the trade-offs associated with each. These approaches vary widely in their suitability depending on polymer type, contamination level, and desired end-use, and understanding their respective strengths and limitations is essential for designing effective and sustainable recycling systems. Following this analysis, we explore the critical role of multidisciplinarity in addressing the complex challenges of plastics recycling, including technical, environmental, economic, and policy/social (TEES) dimensions. Together, these two sections provide the foundation for the final part of this perspective, where we present the spirality framework in detail as a unifying approach that moves beyond traditional loop-based classifications and toward a more integrated, systems-level approach for plastics circularity.

## 3 Current Challenges in Plastics Recycling

The main current recycling pathways are mechanical recycling, and advanced recycling, which includes chemical recycling and plastic dissolution methods.^24–27^ Mechanical recycling relies on heat and physical force to remelt and reprocess plastics, while chemical recycling breaks them down through chemical reactions. Dissolution falls somewhere in between the two, using solvents to selectively dissolve and purify polymers without altering their chemical structure or triggering chemical reactions.

Mechanical recycling, which involves sorting, washing, shredding, melting, and remolding plastic waste into new materials, is the most dominant mode of plastics circularity today.^25^ Polyethylene terephthalate (PET) and high-density polyethylene (HDPE) bottles and containers are among the most commonly mechanically recycled plastics, though in principle, all thermoplastics are suitable for mechanical recycling.^24^ It is a straightforward, less complicated recycling method, outperforming in most instances virgin plastic production and other recycling processes across several economic and environmental metrics.^26–28^

However, the properties of mechanically recycled plastics do not remain the same due to degradation from heat, mechanical stresses, and oxidation during processing, resulting in chain branching and scission, hydrolysis, and molecular weight loss.^25,29–31^ Mechanical recycling of PET resin can lead to color formation and intrinsic viscosity (IV) drop. Color formation arises due to thermal degradation, oxidation, and contamination, leading to yellowing or discoloration of the recycled PET (rPET).^32^ IV drop occurs as polymer chains break down due to hydrolysis and thermal degradation, reducing molecular weight and mechanical properties. To restore rPET quality, solid-state polymerization (SSP) is commonly used, where the material is heated below its melting point (typical temperature range: 200–250 °C) for several hours and under an inert gas flow, which removes condensation byproducts and restores the IV.^33,34^ However, while SSP effectively enhances rPET properties, its environmental impacts can be substantial due to high energy consumption and process emissions. In particular, SSP can contribute significantly to the total GHG emissions of rPET, with values around 0.6 kg CO_2_-eq/kg for food-grade rPET pellets (based on the ecoinvent v3.11 cutoff model data set: “PET pellets, food grade, recycled to generic market for polyethylene terephthalate, bottle grade”).^35^ This accounts for approximately 20% of the total GHG emissions associated with virgin PET bottle-grade production, which ranges from 3.2 kg CO_2_-eq/kg (Europe) to 3.5 kg CO_2_-eq/kg (rest of world) according to the same database.^35^

Such degradation during mechanical recycling affects the molecular structure, and consequently, the material’s mechanical properties, reducing the quality of recycled products (recyclates). For example, studies simulating the mechanical recycling of polymers at the lab scale through repeated shredding and extrusion or injection molding cycles, have observed a significant drop (∼15%) in hardness and instant modulus after the 10^th^ consecutive recycling cycle for HDPE, which increased with additional cycles,^36^ and 50% decrease in strain at break after 10 recycling cycles, while another study^37^ observed a 30% decrease in stress at failure and elongation at break for polypropylene (PP) after 19 cycles.

Additionally, plastics experience further fatigue and property deterioration during use due to environmental stressors. For example, ultraviolet (UV) exposure from sunlight causes photo-oxidative degradation in both single-use plastics, like polyethylene (PE) and PP packaging, as well as in more durable materials like polyvinyl chloride (PVC) pipes. Similarly, humidity accelerates hydrolysis in moisture-sensitive polymers, such as PET and polybutylene terephthalate (PBT). A study tested postconsumer PET bottles under humid and dry conditions over five consecutive recycling cycles.^38^ The results showed that humid specimens lost 73% of their elongation at break and 42% of their tensile strength over these cycles. Stabilizers, such as antioxidants and moisture scavengers, can help mitigate these issues and extend polymer lifespans.^39^ However, they also increase the complexity of plastic formulations, further enhancing the heterogeneity of plastic waste entering the recycling stream, since mechanical recycling does not purify at the molecular scale.^21,39^

Contamination from mixing incompatible plastics or plastics with different compositions and thermal and mechanical exposure profiles and processing backgrounds, as well as the introduction of impurities during waste collection and sorting, are other major factors contributing to recyclates’ degradation.^29^ Compositional variations, combined with continuous degradation, progressively reduce the functionality and quality of recyclates, leading to downcycling and eventually making them unsuitable for further processing or reuse.

Recyclates exhibit pronounced microstructural heterogeneity that evolves with each recycling cycle, yet no standardized methods for assessing their mechanical performance exists. Current mechanical testing guidelines for recycled plastics remain rooted in standards developed for nearly homogeneous polymers. For example, the Association of Plastic Recyclers (APR) Critical Guidance Protocol for HDPE containers^40^ adopts ASTM D638,^41^ suggesting just five or ten specimens per test. Such a small sample size is insufficient when the tensile response of recycled HDPE spans a broad distribution.^29^

To address such limitations, the development of new testing standards that explicitly account for recyclates variability is necessary. First, sampling protocols must be expanded to require significantly more specimens, thereby ensuring statistical convergence of key metrics. Second, specimen geometries should be revisited: gauge sections could be lengthened or multiplexed (e.g., multiple parallel gauge sections) so that local anomalies/heterogeneities are integrated into the overall mechanical response.^42–44^ Third, preparation procedures, e.g., cutting, conditioning, and surface finish, must be standardized to minimize artifactual variability introduced during specimen fabrication. Finally, the analyses and interpretation of the data using statistical methods is crucial for quantifying the variability.^45^ Calculating parameters, such as mean values and standard deviations, would allow for a more robust understanding of the material’s behavior. For example, for materials exhibiting more brittle fracture, considering the use of Weibull statistics can be particularly helpful.^46^ Overall, defining a *heterogeneity index* for recyclates could provide a standardized metric for comparing different batches of recycled polymers and assessing the consistency of their mechanical properties.

Unlike paper recycling which is widely feasible—over 68% of the generated paper and paperboard waste is being recycled today in the US^47^—plastics recycling faces significant challenges in maintaining material quality and purity. During paper recycling, shorter fibers (fines) are eventually removed from the system,^48^ whereas shorter polymer chains in recyclates remain, contributing to material property degradation. Additionally, mixed paper fibers still produce paper, whereas blending different polymers results in an incompatible, heterogeneous material. Paper recycling also benefits from creating a dilute pulp with water, allowing impurities to be washed out,^48^ a process that is not possible in mechanical plastic recycling but can be implemented in plastics’ dissolution.

Diluting recyclates into virgin plastics is a potential, though imperfect, solution to overcome these issues—*“dilution as a solution to circularity”*, paraphrasing a well-known aphorism for pollution. However, this approach comes with the corresponding environmental costs. At the same time, a growing number of research papers^49–53^ are proposing innovative chemistry-enabled pathways for closing the loop in plastics recycling and directly upcycling incompatible polymer blends, using compatibilizers or additives to restore the mechanical properties of recyclates. Yet, the environmental impacts of these chemicals remain poorly understood due to the lack of data and transparency. Manufacturers often do not disclose the specific additives used in their products, making it difficult to assess their environmental impacts, or trace their entry into recycling streams. Moreover, additives can be released from plastics during recycling or recovery processes, potentially ending up in air or water discharges and contaminating the final recycled products. Thus, determining whether recycling truly delivers environmental benefits in such cases remains complex and uncertain.^21,39^

Additional operational challenges hinder the widespread adoption of mechanical recycling, particularly for plastic packaging that comes into contact with food. Residual food contamination requires more intensive washing,^54–56^ involving higher temperatures, chemical washing aids like sodium hydroxide solutions, and an energy-intensive wastewater treatment process due to the high biological oxygen demand in washwater effluents. This further increases the environmental and economic burden of the recycling process, making food-grade plastic recycling more complex and resource-intensive.^54^

Furthermore, many plastic products, such as flexible packaging, consist of multilayer films or polymer composites bonded with adhesives, making them extremely difficult to separate and mechanically recycle.^57^ These layers are designed to provide advanced properties, such as moisture and oxygen barriers, but their complexity requires additional processing steps like delamination or solvent-based separation—methods that are often impractical or unfeasible. Mixed plastic waste presents another challenge due to the variability in material streams. While sorting technologies can identify and separate objects made of different polymers, they cannot delaminate films or effectively handle heterogeneous blends.^58^

Chemical recycling offers a more flexible approach that can process a wider range of complex plastic feedstocks.^25^ It is a rapidly growing method that has gained significant attention over the past decade for its potential scalability. Chemical recycling refers to a suite of technologies that alter the chemical structure of plastic waste by breaking down long polymer chains into shorter molecules through chemical, thermal, or catalytic processes. These shorter molecules serve as the building blocks for new polymers and can be reused as feedstock for producing recycled plastics and other chemicals.^24,59^

Chemical recycling technologies generally fall into two categories: high-temperature processes or thermolysis, and depolymerization methods, also referred to as solvolysis, chemolysis, or remonomerization.^25,27,59^ High-temperature approaches, such as pyrolysis, gasification, and hydrogenation, typically operate above 400 °C and produce a mixture of oils, gases, waxes, and char. In contrast, depolymerization processes, like methanolysis, glycolysis, and hydrolysis, involve milder temperatures (usually below 200 °C) and solvents to break down polymers into monomers or oligomers adding back a small molecule in the reverse of condensation polymerization.

Pyrolysis and gasification convert plastic waste through thermal degradation, with pyrolysis occurring in the absence of oxygen and gasification in limited oxygen conditions.^59^ Pyrolysis is mainly applied to thermoplastics formed via addition polymerization, such as PE and PP, while gasification can process both addition and condensation polymers. These high-temperature processes are often considered “waste-to-energy” recycling technologies, as they typically yield fuels or energy rather than monomers. “Back-to-monomer” systems require highly pure outputs, which are difficult to achieve through pyrolysis or gasification alone. However, pyrolysis oils can be further treated and refined into feedstocks for new plastics, though current applications remain limited.

Depolymerization of plastics in a solvent, often aided by a catalyst, is limited to polycondensation polymers, like PET, polyamides (e.g., nylon), and polycarbonate (PC), breaking them down into their original monomers or related derivatives.^59^ Commonly applied solvents are water, amines, alcohols, and acids. These methods are classified as “back-to-monomer” recycling systems, as they yield well-defined chemical outputs that can be repolymerized into new plastics.

The main advantage of chemical recycling is its ability to produce a wide variety of outputs with virgin-like quality, while also being capable of handling mixed/multilayer plastic waste streams that mechanical recycling cannot process effectively. Despite their potential, chemical recycling technologies face several challenges that hinder widespread adoption.^60^ They require high energy inputs and large, capital-intensive facilities to achieve economic viability.^25,61^ Additionally, these processes are often sensitive to contaminants and additives, which can reduce efficiency and product quality.^62^ Concerns over environmental impacts, scalability, and overall process complexity further limit their competitiveness.^25,27,60,62^ The reliance on chemicals and complex separation systems makes them more costly and currently less environmentally favorable than mechanical recycling. As a result, chemical recycling accounts for less than 1% of all recycled plastics.^25^

Plastic dissolution, also known as solvent-based purification or solvation, is an emerging physical recycling process in which a specific polymer is selectively dissolved from mixed plastic waste using a suitable solvent. This allows the targeted polymer to be separated and recovered in a purified form without altering its chemical structure.^24^ Therefore, this is an advanced recycling process that differs from chemical recycling.

Dissolution offers a promising, tolerant to contaminants recycling approach,^62^ with some studies showing lower^27,28,63^ or comparable^27^ environmental impacts to virgin polymer production. Industrial applications already exist, targeting and separating polymers, e.g., PP,^64^ from mixed or multimaterial waste streams. However, their broader application remains limited due to the need for large volumes of solvents, energy-intensive recovery systems, and challenges in scaling up.^62^ With most systems still at the pilot stage and mechanical recycling remaining more cost-effective, widespread adoption is currently constrained.

## 4 It Takes More than Two to Tango: Multidisciplinarity as a Driver for Sustainable Recycling

Increasing plastics recycling is an important goal, but it can result in performance metrics that fall short of those associated with virgin polymers. For example, as discussed in Section 3, some recycling processes may be highly energy intensive, like SSP,^33,34^ or include additives that pose unintended environmental risks, e.g., potential ecotoxicity impacts of using azidotriazine as a compatibilizer in.^49^ The variability in mechanical properties and the ability of new recycled materials to effectively substitute their virgin counterparts in engineered resin markets is often overlooked. Prominent publications, e.g., refs 49–53, and 65 , often make bold claims about emerging chemical upcycling without fully examining potential trade-offs or limitations. Addressing this challenge requires early multidisciplinary collaboration with experts in life cycle assessment (LCA), techno-economic analysis (TEA), materials science, process engineering, and public policy. Such collaborations can ensure that recycling innovations are not only effective in the lab but also scalable and sustainable in real-world systems, as well as shorten the time frame from ideation to translation and wider adoption.^60,62,66^

A major barrier to evaluating advancements in recycling chemistry is the lack of accessible, transparent, and high-quality data on the life cycle impacts of the tens of thousands of chemicals, e.g., additives and compatibilizers, used to enable polymer recycling.^67^ This data gap hinders sustainability assessments and the development of standard evaluation practices. Closing this gap is critical for informed decision-making and responsible chemical management. Pre-use screening of these substances is essential to ensure a clear understanding of their environmental implications.

An additional challenge lies in outdated LCA data, which often reflects legacy industrial processes and does not account for recent shifts in energy and emissions profiles. For example, polytetrafluoroethylene (PTFE) production involves an intermediate step in which chlorodifluoromethane is generated for the synthesis of the fluorocarbon monomer. This compound is reported to have GHG emissions approximately an order of magnitude higher than those of most virgin polymers.^35^ Over the past two decades, chemical manufacturers have prioritized the effective management of these high-GHG intermediates to reduce emissions. However, the fact that some LCA databases^35^ still do not reflect these changes can result in potentially distorted LCA outcomes for products containing such downstream materials.

Another important consideration in evaluating recycled plastics is cost competitiveness. Current price disparities between recycled and virgin materials are largely driven by economies of scale that favor virgin production. Mature, large-scale processes keep virgin polymer costs low, while recyclates often remain more expensive due to limited supply, contamination, and labor-intensive sorting—much of which still relies on manual methods.^68^ Virgin plastics also offer more consistent quality, making them the default choice for many manufacturers.

However, this landscape is evolving. Legislative instruments such as extended producer responsibility (EPR) and minimum recycled content (MRC) requirements are reshaping market dynamics by creating guaranteed demand for recyclates.^69^ For example, the European Union will require all PET bottles to contain at least 30% recycled content by 2030,^70^ with similar mandates emerging in US states like Washington and New Jersey.^71^ Although these policies may initially raise costs due to supply constraints and stringent quality standards,^72^ they are expected to catalyze investment and innovation in recycling technologies, infrastructure, and supply chains. Over time, these improvements in efficiency, scalability, and material recovery are likely to reduce costs, enhance quality, and help close the gap with virgin plastics—ultimately enabling economic competitiveness and wider adoption of recycled materials.

Encapsulating all these different perspectives in a single study is very challenging and cannot be performed by single teams. Multidisciplinary efforts (e.g., the National Science Foundation Center for Sustainable Polymers^73^ are needed to enable such comprehensive assessments. Developing and evaluating new materials and processes is highly complex, requiring collaboration across various fields.^74^ Given the urgency of addressing plastic waste, timely action and strategic collaboration is needed to ensure that new technologies can be effectively developed and implemented.

## 5 The Spirality Framework as a Way Forward

We propose that the widely used concept of circularity be reframed as spirality, which more accurately reflects the progressive degradation that thermoplastics undergo over multiple recycling cycles. Rather than envisioning polymers as moving through perfectly closed loops, where material quality remains constant, they are instead moving through recycling spirals, where downward movement typically results in some loss of performance due to thermal, mechanical, and oxidative degradation (Figure 2). Each turn of the spiral represents the material’s use and successive mechanical recycling cycle (1, 2, ... n in Figure 2), during which key polymer characteristics, such as IV, tensile strength, and opacity, progressively change. As the material moves through successive spiral turns, it becomes increasingly difficult to be reused in applications that require the same material quality, performance, or purity as the original use, ultimately leading to downcycling. For example, after several recycling cycles, rPET from beverage bottles may no longer meet the purity or mechanical strength requirements for food-grade applications and is instead used in less demanding products, such as textile fibers.^75^

**Figure 2:**
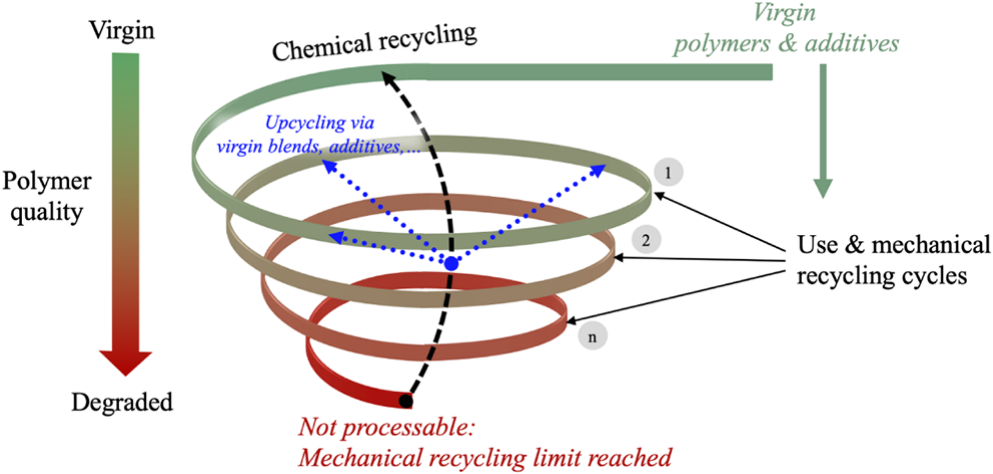
The spirality framework. Green indicates high-quality virgin feedstocks, while red indicates degraded material quality. Virgin feedstocks enter the mechanical recycling system and gradually degrade with each recycling cycle. At each level, it is possible to intervene and restore or preserve polymers’ performance to some extent, either moving upward (upcycling) or retaining the same quality (blue dotted arrows). Eventually, the polymers reach a point where mechanical recycling is no longer viable, requiring chemical recycling to return the material to the supply chain for use in valuable applications.

Movement through the spiral is not necessarily downward. At each level, it is possible to intervene and restore or preserve material performance to some extent, either moving upward (upcycling) or retaining the same quality (blue dotted arrows in Figure 2). Several strategies, such as blending with virgin polymers, addition of compatibilizers and functional additives, and SSP in the case of condensation polymers, can improve or stabilize mechanical properties. Dissolution, where applicable, can be also used to selectively purify target polymers from mixed or contaminated streams without breaking down the polymer chains. These interventions can enable recycled plastics to retain or regain utility across a broader range of applications.

Nevertheless, the feasibility and effectiveness of such interventions depend heavily on the specific material or product type. For instance, multilayer plastic films commonly used in food packaging cannot be mechanically recycled back into the same application due to the immiscibility of their layers; even with compatibilizers, the resulting material is typically nonfood-grade.^57^

Even with the application of such interventions and the mitigation of quality loss across spirals, over time polymer chains undergo irreversible molecular-level degradation through chemical reactions, such as chain scission, oxidation, cross-linking and hydrolysis, as previously discussed (Section 3). These transformations progressively reduce the polymer’s mechanical robustness, ultimately rendering it unsuitable not only for mechanical recycling but for any further material use. At this terminal point—the bottom of the spiral in Figure 2—chemical recycling becomes the most viable option, enabling its upcycling through deconstruction of the polymer into new monomers and other useful chemical intermediates. Some polymers, particularly those synthesized via condensation reactions, such as PET or nylon, allow for relatively efficient monomer recovery, through processes like hydrolysis, methanolysis or aminolysis. Others, especially those produced by free radical polymerization (e.g., PE and PP), require high-temperature thermochemical processes, such as pyrolysis or gasification (see Section 3). These routes yield more complex chemical mixtures that must be further refined and can be integrated into general-purpose chemical supply chains.

Inevitably, given the structure of today’s petrochemical industry, these routes will mix the chemically recycled molecules with those produced from virgin, fossil-based resources, including those destined for use as fuels.^59^ Chemical recycling requires careful tracking of how much carbon from recyclates ends up back in molecules going into chemicals rather than energy, but this is well within our LCA capabilities. While chemical recycling offers a valuable outlet for degraded materials, it is essential that we strive to retain plastics at the higher levels of the spiral for as long as feasible.

LCA and TEA should be integral components of this framework, as they can enable the evaluation of environmental and economic trade-offs at different points along the spiral. This integration allows for the identification of conditions under which maintaining materials at higher spiral levels does not deliver net environmental or economic benefits, compared to transitioning to chemical recycling or to using virgin feedstocks. For instance, as discussed in Sections 3 and 4, the use of additives to extend material usability can introduce significant environmental burdens beyond GHG emissions, potentially offsetting the benefits of continued mechanical recycling. TEA should be also applied in parallel to assess the cost-effectiveness and technical feasibility of such interventions, identifying break-even points by accounting for processing costs, recyclate market values, and application-specific performance requirements.

To illustrate the spirality concept, the use of PET in bottle-to-bottle recycling is considered as an example (Table 1). After its initial use, the PET bottle can be mechanically recycled. Such use and processing cycle will affect the IV of rPET, which typically declines from 0.80–0.83 dl/g (virgin levels) to about 0.70 dl/g.^76^ At the same time, key mechanical properties will deteriorate. For example, tensile yield strength, which is critical for preventing bottle cracking or bursting under pressure, can decrease by approximately 30%. Elongation at break, which reflects the material’s shatter resistance, can also decline significantly.^38^ As shown in Table 1, after this first use and processing cycle, these values approach the lower limits of acceptability for commercial water bottles, which generally require IV ≥ 0.70 dl/g, tensile yield strength ≥ 50 MPa, and elongation at break ≥ 50%. By the second use and recycling cycle, rPET’s IV often falls below the 0.70 dl/g threshold, and its elongation at break drops significantly further, indicating rising brittleness and stiffness levels.^38^ Such property decline eventually causes rPET to breach key industry bottle-grade thresholds.

**Table 1:** Typical Mechanical Property Ranges for Commercial PET Water Bottles^38,77^

Material Characteristics	Bottle-Grade Threshold	Virgin PET Quality	rPET Quality after Mechanical Recycling Cycle: 1	rPET Quality after Mechanical Recycling Cycle: 2	rPET Quality after Mechanical Recycling Cycle: 3
**Intrinsic Viscosity**	0.70 dl/g	0.80–0.83 dl/g	0.70–0.78 dl/g	0.65–0.70 dl/g	<0.65 dl/g
**Tensile Strength**	≥50 MPa	∼55 MPa	∼38 MPa	∼35 MPa	∼30 MPa
**Elongation at Break**	≥50%	>100%	∼80%	∼20%	<15%

IV can be used as a primary metric or proxy to assess rPET quality, since its drop would be linked to the decrease of key mechanical properties. This property degradation in rPET can be addressed through SSP. During SSP, the IV of rPET can be restored or even increased beyond 0.70 dL/g, by treating the material at temperatures between 200 °C and 230 °C for 2 to 8 h.^34^ Both higher temperatures and longer reaction times have been shown to significantly enhance IV. In addition to molecular weight recovery, the crystallinity of the material can also be restored to levels comparable to virgin PET. These improvements contribute to significant enhancements in the mechanical properties of the recycled polymer. Yet, SSP can result in significant environmental impacts (see relevant discussion in Section 3), and even under optimal conditions cannot reverse major degradation by oxidation, color formation or contamination.^33,34^ It can, however, move the rPET back up the spiral to a higher, though still reduced, quality compared to virgin bottle-grade PET, for example to fiber-grade applications, such as carpet manufacturing. While strategies such as SSP, compatibilizers, and blending with virgin PET can extend its usability, these interventions have limits. To determine when such an intervention is justified, both LCA and TEA should be conducted to assess the environmental impacts and economic viability. These analyses can identify the conditions under which it is preferable to restore PET quality versus allowing it to be downcycled. Eventually, issues like inorganic contamination and irreversible degradation (e.g., cross-linking, color formation) will prevent the further reuse of rPET. Then, transitioning to chemical recycling will become the most viable option, allowing for rPET to be returned to chemicals and polymer precursors that are suitable feedstocks for new production. This simple example underscores how spirality, illustrated in Figure 2, can be conceptualized in practice.

The spirality framework applies to all thermoplastic polymers capable of reprocessing, capturing both the decline in material quality and the potential for targeted interventions throughout their recycling lifecycle. While the rate and nature of degradation may vary depending on polymer type, all thermoplastics undergo progressive changes in molecular structure and performance with repeated processing, making the spirality model broadly applicable across polymer classes.

The concept of spirality has been used before in the context of recycling, circular economy, and LCA, but with a different meaning than the one articulated here. For example, Xie et al., 2024 ^77^ use a two-dimensional spiral, where each turn represents a single product life cycle. This visual framework models the progressive flow and dissipation of materials in an infinite-life-cycle system under circular economy conditions. While this approach captures the cascading and time-evolving nature of recycling loops, it does not account for material quality degradation across cycles. Additionally, the term “service equivalents” used to compare recycled and virgin materials is not clearly defined, limiting the interpretability of functional performance across life cycles. Another study^78^ uses the term spirality in the context of recycling PET bottles in China applying a multiple life cycle approach. However, they define it as an economic parameter (the ratio of the value of the recycled material to that of virgin material) not as an indicator of material quality. Ashby et al., 2023 ^79^ introduce the spiral economy as a conceptual extension of the circular economy that explicitly integrates the social dimension. Rather than describing spirals within material use, this spiral economy framework connects social, environmental, and economic systems through dynamic, multidirectional flows. It moves beyond closed-loop models by emphasizing social inclusion, learning, open innovation, and the creation of social value and capital. This interpretation differs significantly from ours. Valero et al., 2023 ^80^ also use the term spiral economy, with spirality representing the imperfect, ongoing flow of materials through multiple life cycles. Unlike the ideal closed loop of circularity, the spiral reflects a reality where each cycle involves some material loss due to thermodynamic and practical limits. It symbolizes partial recovery, not total closure, emphasizing the need to design products for repeated use, repair, and eventual recycling, while acknowledging that some losses are inevitable. While this interpretation of spirality aligns conceptually with ours, it remains largely theoretical and abstract. Unlike our work, it does not provide practical applications related to recycling methods or recycled materials, nor does it address material quality degradation across cycles.

While the spirality framework offers a more realistic view of plastics recycling, it is not without limitations. A central challenge is the lack of high-quality data on how polymer properties change over multiple recycling cycles and when blended with virgin materials. This limits our ability to quantitatively assess material degradation, recovery potential, and the effectiveness of interventions across spiral pathways. Measuring spirality, in this context, means understanding the relationship between the number and type of recycling processes a material undergoes and the resulting changes in its functional properties. Rather than introducing new metrics, such as a “spirality index”, we advocate for evaluating spirality using established system-level TEES metrics. Proliferating new circularity- or sustainability-focused indices risks complicating decision-making without delivering clearer system-level insights. By embedding spirality into existing metrics and frameworks like LCA and TEA, we can assess whether spiral material flows improve system performance or simply add complexity. This approach also highlights the need for more robust data collection, particularly for recyclates and blended materials, to capture the variability and trade-offs inherent in real-world recycling systems.

Our articulation of spirality is novel as it draws attention to a critical yet often overlooked aspect of reuse and recycling systems: the progressive degradation of material properties—both physicochemical and functional—that determines whether a material can be reused in the same or alternative applications or markets. While the concept of a reuse hierarchy already exists, it has not previously been expressed through the geometric notion of spirality, nor has it been applied to materials for which quality and application requirements can be quantitatively assessed. Our spirality framework offers a more realistic and dynamic lens for evaluating plastic recycling pathways, capturing both the potential for material recovery and the constraints imposed by property degradation and processing limits. It emphasizes the need for system-level thinking in material design, policy, and recycling infrastructure, shifting the narrative from idealized closed loops to more adaptive and pragmatic resource flows. It can serve as a practical framework for sustaining material value and performance across cycles. Lastly, it underscores the need to align three development pathways to enable effective interventions and avoid unintended trade-offs: a) novel chemistries (e.g., new polymers and additives that resist degradation or enable targeted repolymerization), b) high-throughput mechanical testing for robust recyclate property data, and c) advanced assessment frameworks (e.g., expanded LCA and TEA frameworks with transparent, quality data and assessment of relevant TEES metrics).
